# Effectiveness of Ultrasound Cardiovascular Images in Teaching Anatomy: A Pilot Study of an Eight-Hour Training Exposure

**DOI:** 10.3390/ijerph19053033

**Published:** 2022-03-04

**Authors:** Mariam Haji-Hassan, Tudor Călinici, Tudor Drugan, Sorana D. Bolboacă

**Affiliations:** Department of Medical Informatics and Biostatistics, Iuliu Hațieganu University of Medicine and Pharmacy Cluj-Napoca, Louis Pasteur Str., No. 6, 400349 Cluj-Napoca, Romania; mariam.haji-hassan@umfcluj.ro (M.H.-H.); tdrugan@umfcluj.ro (T.D.); sbolboaca@umfcluj.ro (S.D.B.)

**Keywords:** cardiac anatomy training, medical students, hand-held ultrasound, cadaveric images

## Abstract

The present study aimed to evaluate the students’ progress in evaluating ultrasound (US) and cadaveric cardiac images and long-time retention of information. First-year medical students were invited to participate in four two-hour online lectures during one week voluntarily. The students were trained to recognize cardiovascular anatomical structures on US and cadaveric images during the intervention. The participants’ abilities to identify specific anatomical structures were tested before, immediately after and six months after the training. A group of second-year students without US training participated as a control group and filled the same test once. Ninety-one first-year students agreed to participate, and forty-nine completed all three tests. The performances in the correct identification of cardiovascular structures on the US images significantly improved after the training but significantly decreased after six months. In the intervention group, the accurate identification of cardiovascular structures was significantly higher on cadaveric images (80% vs. 53%, *p*-value < 0.0001, *n* = 91 at post-training; 70% vs. 33%, *p*-value < 0.0001, *n* = 49 at 6 months after training). The correct answers percentage score in the control group varied from 6.7% to 66.7% for US cardiovascular anatomical without a significant difference than the intervention group (*p*-value = 0.7651). First-year students’ knowledge of heart US anatomy proved less effective than cadaveric images, significantly improved after training and decreased over time, indicating the need for repetition reinforcement.

## 1. Introduction

Ultrasound examination has become widely used by most medical specialties [1]. However, not all medical schools have integrated ultrasound (US) training into their curricula [2]. Although it is usual to start teaching US to students during clinical years, some studies promote the idea that preclinical years are more appropriate to understand anatomy and physiology [3,4]. Positive experiences of the US used in teaching anatomy were previously reported on upper limb and lower limb anatomy [5] and abdominal anatomy [4,6]. The use of US in learning anatomy had at least two advantages: move the student’s visual understanding from 2D (cadaveric images) to 3D (real-time imaging of a living patient) and provide skills in being able to scan a patient that would be useful in clinical practice [7].

One constant among the heterogeneous literature is the students’ reaction to the US training. They have consistent positive responses to such training and are more likely to incorporate US in their future medical practice [8,9]. Allsop et al. [10] recommend using US during anatomy teaching, stating benefits such as a better understanding of the dynamic nature of living anatomy and improved motivation to study. Therefore, the integration of US in the medical school curriculum is both feasible and beneficial to medical students. Researchers support the idea that US imagery is as effective as cadaver dissection in teaching anatomy [11,12] and recommend integrating US training in medical curricula [13]. However, the integration of US training in anatomy courses is reported only by 8/46 universities in Europe [14], although US training integration in medical curricula is well received by the students [4,15,16].

When asked, students expressed their desire to learn US during preclinical years. However, the most challenging problem is finding a sufficient number of trainers to maintain a low student-to-instructor ratio [4], even though there is evidence that after a few hours of training, anatomists could be as effective as clinicians in teaching US during anatomy lessons [17]. Limited evidence exists concerning long-term retaining of information and validated tools to assess students and US training programs.

Ultrasound device miniaturization led to the appearance of hand-held US devices. They have the advantages of increased accessibility and lower cost, although the image quality is not always very qualitative. Kapur et al. used such a device (e.g., General Electric’s Vscan) to teach students abdominal anatomy, receiving positive reactions from students and tutors alike [18].

Even if heart US is widely perceived as technically challenging, and satisfactory image quality is often challenging, especially with a hand-held device, our study aimed to evaluate the students’ progress in reading US and cadaveric cardiac images and long-time retention of information.

## 2. Materials and Methods

### 2.1. Ethics and Consent

The present study was approved by the Ethics Committee from “Iuliu Hațieganu” University of Medicine and Pharmacy (approval no. 264/30 June 2021). The acknowledgment to those who donated their bodies to science and their families that allowed obtaining the cadaveric cardiac images was performed following the recommendation from anatomical journal editors [19].

The participation was voluntary, and the student’s decision had no repercussions on the knowledge evaluation at the anatomy exam.

### 2.2. Participants Recruitment and Educational Intervention

First- and second-year French section medical students at the Faculty of Medicine, Iuliu Hațieganu University of Medicine and Pharmacy Cluj-Napoca were invited to participate in four two-hour online classes. The educational intervention took place in the same week and was delivered by the same teacher for all participants. The study was conducted in the 2020–2021 academic year.

The students were trained to recognize cardiovascular anatomical structures on cadaveric and US images.

### 2.3. Test Structure and Data Collection

The same examiner obtained all US images using a hand-held ultrasound (Butterfly iQ+, Butterfly Network, Inc. Guilford, CT, USA). All US images were collected from the same healthy subject. The acquisition of the US cardiac images was made following the current guidelines: four-chamber view (apical view) that allow visualization of all chambers; the subxiphoid view that shows four chambers and aortic valve; the parasternal short axis used to assess tricuspid, pulmonic and aortic valves; and the parasternal long axis that shows coronary sinus and aorta, etc. [20].

A test was designed to evaluate the students’ ability to identify cadaveric and US images’ main cardiac structures. Ten cadaveric images and 15 US images evaluating the same anatomical structures (cardiac chambers—atria and ventricles, interatrial and interventricular septa, mitral valve, tricuspid valve, aortic valve, aorta, papillary muscles) were included in the test. The same structures from different US views were used, resulting in more US than cadaveric images.

The first-year students who consent to participate took the pre-training test (before the lectures) and the post-training test (at the end of the week when educational intervention was taught). The first-year students as participants were asked to take an additional test 6 months apart from the post-training to assess the long-term retaining of information.

The second-year students in the same academic year and the first-year students were asked to act as the control group for the long-term test of the first-year students. The second-year students did not receive training in US image interpretation, and the tests referred to the cardiovascular anatomy material learned one year earlier.

**Figure 1:**
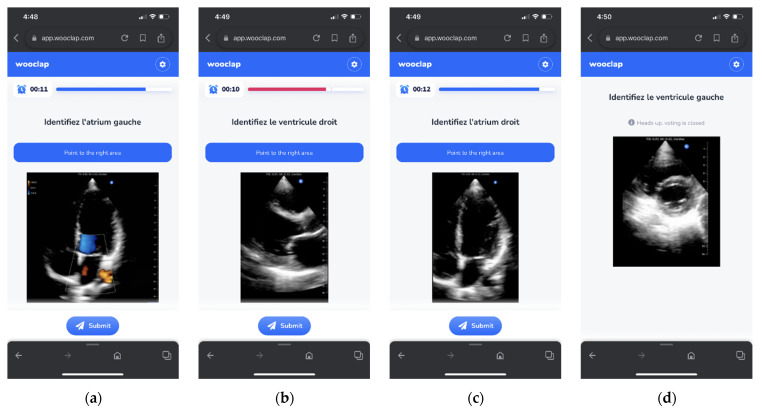
Wooclap US images test by example: (**a**) apical 4-chamber view with color Doppler—identify the left atrium; (**b**) parasternal long-axis view—identify the right ventricle; (**c**) apical 4-chamber view—identify the right atrium; (**d**) short-axis view at the level of papillary muscles—identify the left ventricle.

The students took all the tests using the Wooclap platform (Wooclap, Chasse, Belgium) (Figure 1). All students were previously trained in using Wooclap. The maximum score for each test (cadaveric and US) was 100% and was achieved whenever appropriate anatomical structures were identified. The students had 15 s to answer each question.

### 2.4. Statistical Analysis

The Q–Q plots and Kolmogorov–Smirnov test of the percentage of correct scores were used to assess the assumption of normality. All scores were expressed as the median and interquartile range (25th percentile to 75th percentile) and {min to max} values.

Differences within the intervention group over time (before and after the learning intervention) were analyzed with the Friedman test for all three tests and the Wilcoxon Matched Pairs Test for pairs comparisons. Interpretation of US images scores differences as pre-training vs. post-training and 6-months after in the intervention group were compared to the control group and verified with the Mann–Whitney test. Data were collected and stored in Microsoft Excel and analyzed using TIBCO Statistica, v. 13.5 (TIBCO Software Inc, Palo Alto, CA, USA). Graphical representations were constructed following the recommendations [21]. Statistical significance was defined as *p*-value < 0.05.

## 3. Results

**Figure 2:**
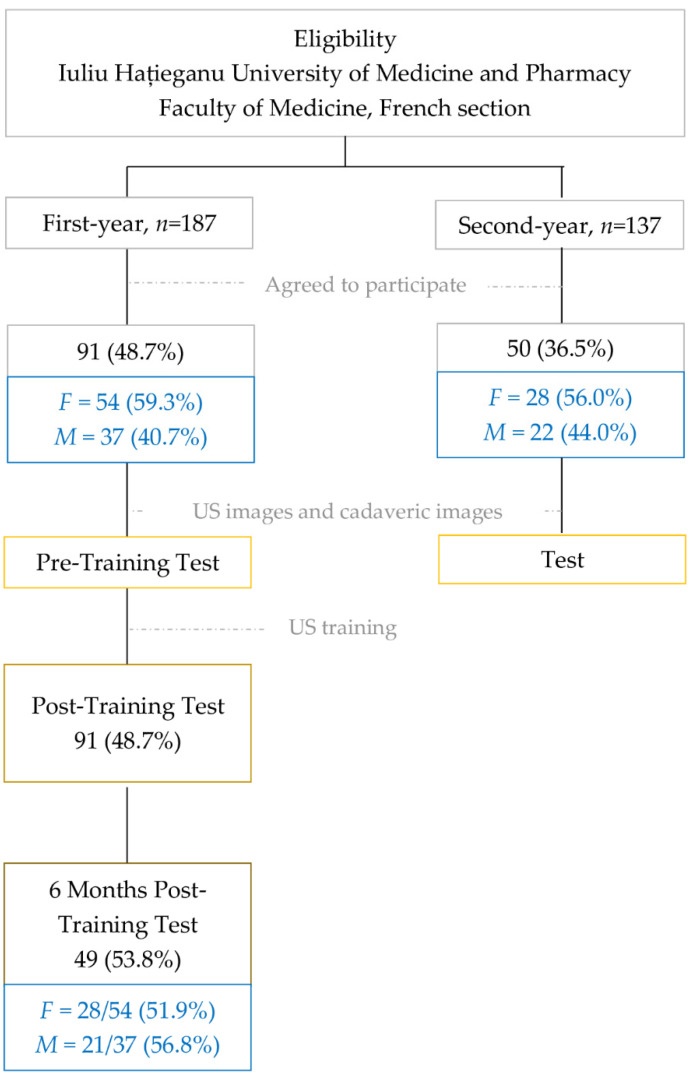
Study flowchart: from eligibility to analysis.

Ninety-one first-year students out of 187 eligible agreed to participate in the training intervention group. Fifty second-year students also agreed to participate in the evaluation of their knowledge one year after receiving the standard cardiovascular anatomy cadaveric training. The participants from the intervention group were tested before US training, after US training and 6-months after the intervention to evaluate the training efficacity. The control group, the second-year students with no US training, were assessed only once. The flowchart of participants’ routes in our study is presented in Figure 2.

No significant differences were observed regarding the gender of participants in the US training intervention and the control group (Chi-squared test: χ^2^ = 0.15, *p*-value = 0.7005).

The correct identification of cardiovascular anatomical structures significantly increased after US intervention, and it is reflected in US and cadaveric images but decreased at the evaluation of 6 months (Table 1).

**Figure 3:**
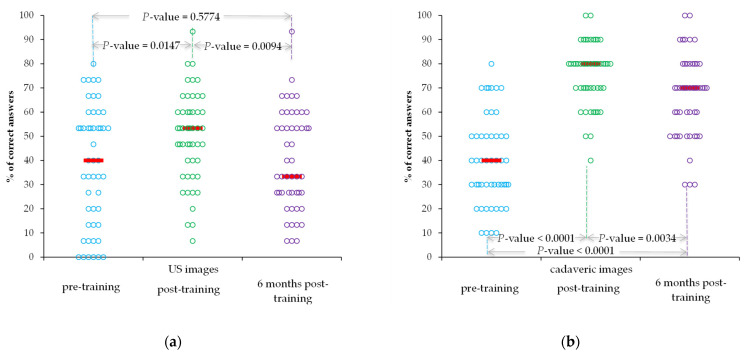
Performances over time regarding the correct identification of cardiovascular structures by students who sit to all three tests: (**a**) US images; (**b**) cadaveric images. Horizontal lines are the median values, and the circles are raw data.

All 91 first-year students participated in pre- and post-training tests, and 49 participated in all tests. The performances in the correct identification of cardiovascular structures on the cadaveric and US images significantly improved after the intervention but significantly decreased six months after intervention (Figure 3a,b).

**Figure 4:**
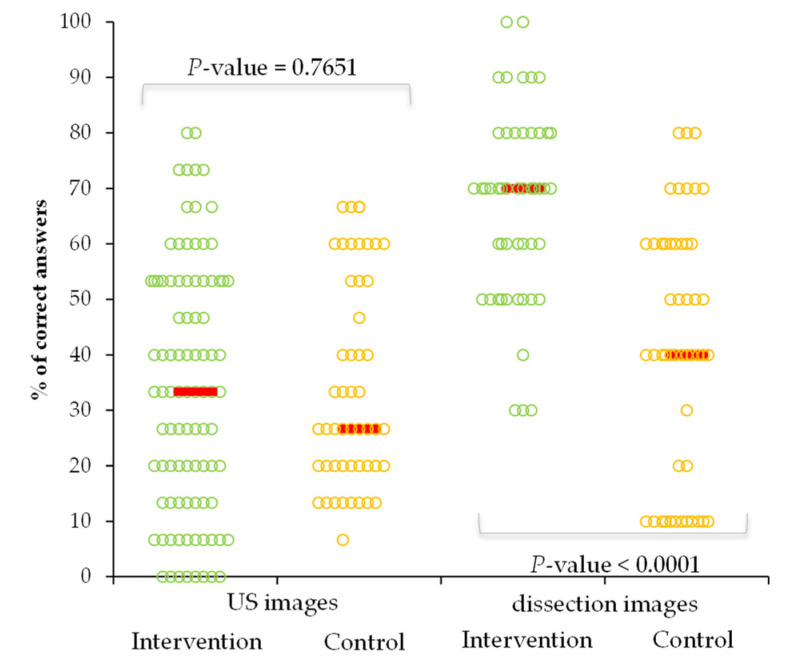
Performances in the correct identification of cardiovascular images by groups. (US = hand-held US images) Horizontal lines are the median values, and the circles are raw data.

The % score of correct answers in the control group varied from 6.7% to 66.7% for US cardiovascular anatomical images with a median of 26.7% (IQR = (20 to 53.3)) and from 10% to 80% (median = 40 (20 to 60)) on cadaveric images. Similar performances were observed when the intervention group was compared to the control group regarding the US images, but with better performances regarding the identification of cardiovascular anatomical structures on cadaveric images (Figure 4).

## 4. Discussion

Our results demonstrated that students improved their ability to recognize anatomical structures on US images early after the limited training sessions but did not reach the level achieved on cadaveric images. The skills decrease over time, are higher on US images than cadaveric images and are more accentuated over time (small scores on the control group), stressing the need for continuous training.

The American Academy of Emergency Medicine encourages US training programs for medical students [22]. The most appropriate time to introduce students to US notions and the optimal duration of training is a debated subject. Even in the first year, medical students show interest in learning US and can assimilate US notions [23]. In our study, 8 h of training were sufficient to determine a significant improvement in recognizing anatomical structures on cardiac US images. However, continuous training seems to be critical, as knowledge retention drastically diminishes over time. The results of our study demonstrated that the ability to interpret US images returned to the baseline (pre-training) after six months (Table 1).

Mid- and long-term retention of information is a matter of continuous interest. Menegozzo et al. demonstrated on medical students no significant difference between post-training taken at one month and three months after training, with only a mild decrease in correct answers over time [24]. Mizubuti et al. demonstrated in a study conducted on residents a negligible reduction in the exam scores 6 months after completing a cardiac US training program, despite personal perception on decreased knowledge after six months [25].

Our findings demonstrated that a short duration of training is effective in the short-term (Table 1 and Figure 3). Town et al. found residents’ knowledge of US increased immediately after a 2.5 h US workshop but significantly decreased in a follow-up 12-month assessment [26].

Griksaitis et al. found similar results when students interpreted cadaveric and US images using a conventional US device [11]. In our study, students performed better when identifying anatomical structures on cadaveric images than US images (Table 1 and Figure 3). Ultrasound images pose a relative difficulty for students, and beginners need optimal image quality to understand US anatomy better. Image quality is generally considered to be lower on hand-held US devices than on their high-end counterparts [27]. This disadvantage needs to be weighed against the lower price and increased availability of hand-held devices [28].

One way to minimize the inconveniences characterizing hand-held devices is to use those brands that have been benchmarked against larger, cart-based devices. While their cost can be higher than some of the cheapest US devices (but still under the USD 10,000 mark), their image quality reportedly resembles what would be expected from a high-end US device [29].

In the near future, Artificial Intelligence (AI) is expected to assist novices in the interpretation of US images, with companies developing deep learning algorithms that can make measurements on US images with less variability than manual measurements performed by expert sonographers [30]. The US examination of the heart is time-consuming, operator dependent and subject to errors [31], but it is a low-cost and non-invasive examination [32]. Incorporating AI and deep learning into hand-held US devices can potentially assist the students in learning how to obtain a quality image and how to interpret US heart images.

### Study Strengths and Limitations

A rigorous design was applied in our study to ensure the validity and reliability of our results. A structured test was used to evaluate students’ abilities to identify cardiac anatomic structures on US and cadaveric images. The same test was used at all stages of the study (pre-training, post-training and 6-months post-training) and was administrated to all participants to ensure the study’s validity and reliability. Furthermore, continuous evaluation was performed to assess the students’ achievements, which allows making reliable decisions about our educational intervention. The educational intervention, including training and assessment, took place online due to the COVID-19 pandemic, ensuring our study’s cost-effectiveness. One educator trained all participants simultaneously, and the same trainer assessed the students’ educational achievements to minimize the inter-trainer bias and the educational intervention costs.

Besides its strengths, our study has some methodological limitations. First, one cohort of students from a single academic program with high heterogeneity regarding cultural and previous education context was evaluated. Students admitted to the French section at Iuliu Hațieganu University of Medicine and Pharmacy come from different parts of the world. Those who agreed to participate may not appropriately represent the target population. Second, the number of students included in the study was small, and the participation in the 6-months post-training test was limited. The inclusion of medical students from all programs in the university and from all sections could enlarge the pool of eligible students, and more appropriate motivation could appropriately reflect the reality. Third, the allocation to the intervention was convenient. A random assignment to the intervention could reflect more appropriately the effectiveness of the US training in the identification of cardiovascular anatomical structures and its role in teaching cardiac anatomy. Forth, due to the COVID-19 pandemic, the US learning sessions took place online, limiting the tutor’s interaction with students and not allowing hands-on training. Given the possibility of performing the cardiac US examination, the students could be more motivated to participate in all follow-ups evaluations and thus increase the power of the study. The US examination of the cardiovascular system is challenging, and it would be interesting to evaluate the efficacy of US anatomy training for other systems.

## 5. Conclusions

Our results demonstrated that the first-year students’ knowledge of heart ultrasound anatomy did not reach the level achieved on cadaveric images, so identification of ultrasound anatomy was not as effective as cadaveric anatomy. The US recognition of heart anatomical structures significantly improves after brief training but decreases over time, showing the lack of information preservation over time. The skills in identifying heart anatomical structures decrease over time and are more accentuated on US images than on cadaveric images, stressing the need for continuous US training to ensure preservation.

## Figures and Tables

**Table 1:** Summary of cardiovascular anatomy test results of cadaveric and US images on the intervention group.

	Metric	Pre-Training	Post-Training	6 Months Post-Training	*p*-Value
US images test		33.3 (13.3 to 53.3) {0 to 80} 40 (13.3 to 53.3) {0 to 80}			
All participants (*n* = 91)	median (Q1 to Q3)		53.3 (40 to 66.7)		<0.0001 *
	{min to max}		{6.7 to 93.3}		
All tests (*n* = 49)	median (Q1 to Q3) {min to max}		53.3 (40 to 60) {6.7 to 93.3}	33.3 (26.7 to 53.3) {6.7 to 93.3}	0.0397 **
Cadaveric images test		30 (20 to 50) {10 to 80} 40 (30 to 50) {10 to 80}			
All participants (*n* = 91) All tests (*n* = 49)	median (Q1 to Q3) {min to max} median (Q1 to Q3) {min to max}		80 (70 to 80) {20 to 100} 80 (70 to 80) {40 to 100}	70 (50 to 80) {30 to 100}	<0.0001 * <0.0001 **
*p*-value US vs. cadaveric images test		0.6160 * (a)	<0.0001 * (a)	<0.0001 * (b)	data

* Wilcoxon Matched Pairs Test; ** Friedman test; (a) comparison was made for all participants; (b) the test was performed for the sample of 49 participants who performed all three tests (pre-training, post-training and 6 months post-training).

## Data Availability

The presented data will not be publicly available until the associated Ph.D. thesis is published. Raw data can be obtained upon request addressed to the first author.
